# In Vitro Regeneration of *Stevia rebaudiana* Bertoni Using Somaclonal Variation as a Tool for Genetic Diversification

**DOI:** 10.3390/genes16101203

**Published:** 2025-10-14

**Authors:** Magdalena Dyduch-Siemińska, Jacek Gawroński

**Affiliations:** Department of Genetics and Horticultural Plant Breeding, Institute of Plant Genetics, Breeding and Biotechnology, University of Life Sciences in Lublin, Akademicka 15 Street, 20-950 Lublin, Poland; magdalena.dyduch@up.lublin.pl

**Keywords:** DNA polymorphism, indirect organogenesis, plant tissue culture, SCoT, somaclones

## Abstract

Introduction: *Stevia rebaudiana* Bertoni has recently gained significant attention due to the presence of intensely sweet yet low-calorie steviol glycosides (SGs) in its leaves, making it a promising natural sugar alternative with applications in the food, pharmaceutical, and cosmetics industries. The primary goal of this study was to determine whether generating somaclonal variation from plant material obtained by indirect regeneration results in further genetic changes identifiable using the SCoT marker (Start Codon Targeted). Methods: In the first stage, callus tissue was initiated from first-generation somaclones on MS medium supplemented with 4.0 mg/L 6-benzylaminopurine (BAP), 2.0 mg/L 1-naphthaleneacetic acid (NAA), and 2.0 mg/L 2,4-dichlorophenoxyacetic acid (2,4-D). Their morphogenetic potential was analyzed on four media with different BAP and Kinetin concentrations. Donor plants, first and second generation somaclones, were also analyzed for genetic diversity using SCoT markers. Results: All first-generation somaclones demonstrated a very high callus initiation capacity, ranging from 95 to 100%. It was found that for most of the studied somaclones, the greatest number of shoots were developed by explants grown in a medium supplemented with 0.5 mg/L BAP and 0.25 mg/L Kin. The studied group of somaclones exhibits a high degree of polymorphism (55.2%). The analysis of genetic similarity of somaclones presented in the form of individual dendrograms indicates that in most cases, greater genetic diversity was revealed as a result of indirect regeneration in the first generation of somaclones compared to the second. Indirect organogenesis allows for the production of subsequent generations of genetically unstable somaclones, creating the potential for obtaining new phenotypic variants useful in plant breeding.

## 1. Introduction

*Stevia rebaudiana* Bertoni (*S. rebaudiana*), a member of the *Asteraceae* family, has gained importance in recent years due to its health-promoting properties and potential as a natural sweetener. The steviol glycosides (SGs) contained in its leaves, characterized by high sweetness yet low caloric value, constitute an attractive alternative to traditional sugar, making stevia the subject of intensive scientific research in the context of the food, pharmaceutical, and cosmetics industries [1]. The stevia genome is diploid (2n = 22), with a heterozygosity index of 0.43%, indicating its high stability. Stevia’s genetic material contains 73.11% repeated sequences, such as microsatellites and transposons, which may be involved in gene regulation and expression [2]. Although the natural form of stevia is diploid, studies have shown the presence of polyploids in this species, such as triploids (2n = 33) and tetraploids (2n = 44). These mutations can arise spontaneously or be induced artificially, for example, with colchicine [3]. Polyploids exhibit morphological and qualitative variability, which may be important in breeding plants with desirable traits [4]. Genetic manipulations contribute to changes in the size and structure of leaves, stomata, and pollen. For example, tetraploids are characterized by larger, thicker leaves and higher chlorophyll content, which may translate into higher steviol glycoside production [3].

In 2020, O’Neill and Pirro [5] sequenced DNA from a commercially grown seedling using the Illumina HiSeq platform. They obtained a total sequence length of 411,383,069 base pairs and identified 24,994 genes. In recent years, most of the genes involved in the biosynthetic pathway of the main steviol glycosides have also been identified and characterized [2]. The high variability in observations regarding their accumulation highlights the complexity of a wide range of interacting biotic and abiotic factors underlying their cumulative effects on plant development. It is known that steviol glycoside accumulation is strongly dependent on ontogeny, photoperiod, as well as factors such as genotype, nutrients, temperature, and irradiance during field production. However, to truly verify the reliability of the influence of the above-mentioned factors on SG synthesis for the *S. rebaudiana* species, it is necessary to include a wider range of genotypes in the studies, preferably those characterized by greater fluctuations in the rebaudioside A to stevioside ratio. Therefore, there is a constant need to develop new stevia cultivars with diverse steviol glycoside profiles, intended for research and commercial applications [6]. Although stevia is naturally propagated from seeds, due to its poor germination capacity, generative propagation is not a common method for its commercial production [7]. A safe alternative that allows not only the propagation of this species but also the development of new stevia genotypes is plant tissue culture—a subdiscipline of modern biotechnology. The main goal of in vitro techniques is the mass production of plants using even a single cell. Micropropagation under sterile conditions utilizes the morphogenetic capabilities of plant cells to quickly obtain plants in large (industrial) quantities. Plants obtained through direct organogenesis are genotypically and phenotypically homogeneous and disease-free, and the primodia of new organs develop directly from tissue taken from the mother plant, which serves as the source of primary explants. Obtaining plants homogeneous in terms of chemical composition and steviol glycoside content is crucial for industrial stevia cultivation, particularly with respect to the stability of steviol glycoside production and the standardization of the plant material obtained from it [4,8,9,10]. In vitro cultures also enable the improvement or induction of new traits in the created cultivars [11]. This is achieved through a method called indirect organogenesis, in which callus tissue first develops, and only then new organs form and differentiate from it [12]. This method takes advantage of the morphogenic properties of callus, which is a wound-related, genetically unstable tissue. Inducing organogenesis through this route can result in somaclonal variability within the obtained plants, which may also differ genetically and phenotypically from each other and from the parent plant. The occurrence of somaclons is influenced by genetic factors, epigenetic culture conditions, and oxidative stress resulting from free radicals. At the molecular level, variability manifests itself through changes in DNA methylation, DNA strand breaks, point mutations, and chromosomal aberrations [13]. Chromosomal rearrangements play a significant role in generating genetic diversity. Point changes in the DNA sequence, consisting of single-nucleotide substitutions, can affect the structure and function of proteins derived from these genes. Furthermore, changes such as translocations, insertions, or deletions of specific DNA fragments can disrupt gene function and expression, also leading to the emergence of somaclonal variants [14]. In addition to genomic changes, epigenetic phenomena also influence the development of somaclonal variability [15].

The primary goal of this study was to determine whether generating somaclonal variation from plant material obtained by indirect regeneration results in further genetic changes identifiable using the SCoT marker (Start Codon Targeted) system. Furthermore, an analysis of the morphogenetic potential of the obtained regenerants was conducted.

## 2. Materials and Methods

### 2.1. Plant Material

**Figure 1:**
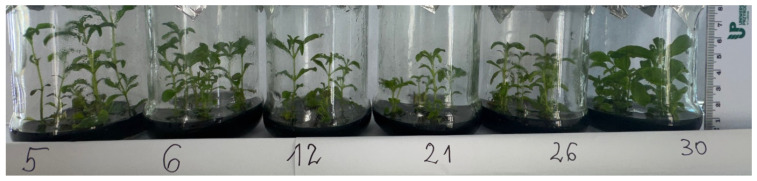
Somaclones of the first generation were used to initiate callus tissue.

This study was conducted on stevia plants (*Stevia rebaudiana* Bertoni) from the in vitro culture laboratory of the Department of Genetics and Breeding of Horticultural Plants. Plants obtained through in vitro micropropagation served as a source of explants. This material was used at the Department to conduct experiments within research projects. The stevia plants used in this study were somaclones obtained through indirect organogenesis (first generation of somaclones) [16]. Based on the differential genetic distance between the somaclones and the Rebaudioside A/Stevioside Ratio, somaclones designated as 5, 6, 12, 21, 26, and 30 were selected for analysis (Figure 1).

### 2.2. Callus Initiation and Generation of Somaclone Second Generation from Leaf Explants

**Figure 2:**
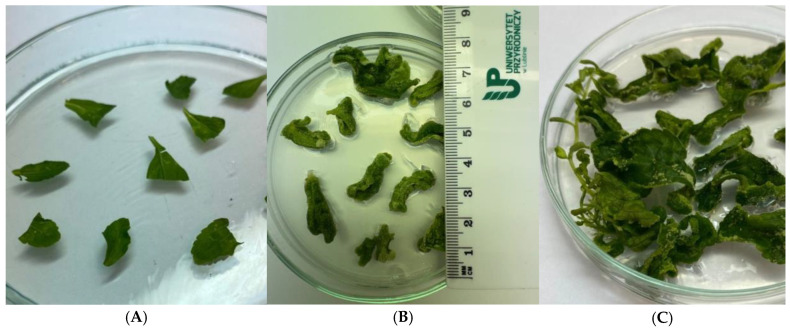
Leaf explants placed on MS medium (**A**) on the day of culture initiation, (**B**) after 4 weeks, (**C**) after 8 weeks.

The callus initiation process began with the collection of leaf explants from the selected *S. rebaudiana* Bertoni plants (mentioned above). The leaves were cut off and divided into fragments, and the explants were transferred to Murashige and Skoog (MS) medium [17], 10 explants per plate and 10 plates per genotype (Figure 2A). The MS basal solid medium was prepared by combining all stock solutions according to the formulation described by Murashige and Skoog. The medium was supplemented with plant growth regulators—4.0 mg/L 6-benzylaminopurine (BAP), 2.0 mg/L 1-naphthaleneacetic acid (NAA), and 2.0 mg/L 2,4-dichlorophenoxyacetic acid (2,4-D), 3%sucrose, 0.8% Difco Bacto-agar. All chemicals used in this study were purchased from Merck (Darmstadt, Germany). The pH of the medium was adjusted to 5.8 using 1 N HCl or 1 N NaOH. The Petri dishes were kept in the phytotron under controlled environmental conditions: temperature 21 ± 2 °C, 16 h light and 8 h dark conditions, approximately 3000 lux light intensity. After 8 weeks, the potential of the callus tissue obtained on the primary explants was characterized by assessing the morphogenetic capacity of the callus and the number of regenerants.

### 2.3. Culture Stabilization

After the regeneration stage, the resulting plants were separated from the callus tissue and placed on MS medium without growth regulators. Each second-generation somaclone was represented by five microplants. The purpose of this stage was to allow shoot morphological development. Furthermore, leaves were collected from each microplant of each somaclone and from the parent plants (somaclone first generation), donor plants (the plant from which the first generation of somaclones was derived—O), and used for DNA extraction.

### 2.4. Analysis of the Morphogenetic Potential of Regenerants

The microplants obtained in the previous step were divided into nodal fragments. They were then placed on media prepared based on MS medium with the addition of phytohormones, the composition of which is presented in Table 1. The aim of this step was to select the optimal composition of the medium for propagating the regenerants. The medium was prepared in jars containing 30 mL of medium. Five nodal explants of each somaclone were placed in a jar, obtaining combinations from 5/1 to 5/5 for somaclone 5, combinations from 6/1 to 6/5 for somaclone 6, combinations from 12/1 to 12/5 for somaclone 12, combinations from 21/1 to 21/5 for somaclone 26, combinations from 26/1 to 26/5 for somaclone 30, and combinations from 30/1 to 30/5 for somaclone 30. The first digit denoted the somaclone number from which the regenerants were derived, and the second digit denoted the regenerant number. The experiment was set up in triplicate. After 6 weeks, the number of shoots, shoot length, and number of nodes were analyzed. A one-way analysis of variance (ANOVA) was performed for each trait, and the significance of differences between means was assessed at α ≤ 0.05 using Duncan’s test.

### 2.5. SCoT Markers—Genotype Analysis

The methodology has been described in detail by Siemińska et al. (2024) [16]. Briefly, genomic DNA was extracted from all samples using the cetyl trimethyl ammonium bromide (CTAB) method as outlined by Doyle and Doyle [18]. To conduct the genetic analysis of *S. rebaudiana* genotypes, PCR conditions were optimized specifically for the tested SCoT markers. The optimization process focused on determining the optimal concentration of magnesium ions, a critical factor influencing DNA polymerase activity and amplification efficiency. This step allowed for the selection of primers that consistently produced stable amplification patterns with clearly resolved bands. Each 10 µL PCR reaction contained water, 1× reaction buffer, 1.5 mM magnesium chloride, 1 µM dNTPs, 0.5 U of Taq DNA polymerase (NZYTech, Lisboa, Portugal), 0.8 µM primer (Genomed S.A., Warsaw, Poland), and 25 ng of genomic DNA. The PCR protocol included 35 cycles, each consisting of denaturation at 94 °C for 1 min, primer annealing at 50 °C for 1 min, and extension at 72 °C for 2 min. This was preceded by an initial denaturation step at 94 °C for 3 min. PCR products were subjected to electrophoresis on a 1.5% agarose gel containing 0.05 μL∙mL^−1^ ethidium bromide. Visualization was performed under UV light, and images were captured using the GeneSnap version 7.09 (SynGene, Cambridge, UK) gel documentation system. To estimate molecular weight, NZYDNA Ladder VIII (NZYTech, Lisboa, Portugal) was used. Only clear and reproducible SCoT fragments were scored based on gel photographs. Bands observed across the tested genotypes were recorded as present (1) or absent (0), representing polymorphic profiles; specific bands unique to individual genotypes were also noted. Poorly visible or ambiguous bands were excluded from further analysis. Cluster analysis was performed using the UPGMA method (Unweighted Pair Group Method with Arithmetic Mean), as implemented in the PAST 3 software [19]. Genetic pairwise similarities between the analyzed genotypes were calculated using the similarity index (SI) based on Dice’s coefficient, following the Nei and Li approach [20], also within the PAST environment [19].

## 3. Results and Discussion

### 3.1. Callus Initiation from Leaf Explants and Obtaining Regenerants by Indirect Organogenesis

When optimizing stevia breeding, emphasis is placed on rapidly obtaining improved genotypes that can yield a large amount of raw material containing fewer substances responsible for bitterness. This makes stevia more readily available to consumers and more readily used as an alternative to sugar, which may prove valuable in combating lifestyle diseases, including type II diabetes [8,16]. Plant tissue cultures are a tool that can be useful in achieving the aforementioned breeding goals. The selection of the primary explant is a key element in inducing somaclonal variation, leading to plants with new traits. To achieve the highest possible level of variation, differentiated tissues such as leaves and stems, which are more susceptible to genetic alterations during culture than meristematic tissues, should be selected as explants. Another crucial approach involves favoring indirect organogenesis over direct methods, as indirect organogenesis typically involves a callus phase associated with prolonged cell proliferation and extended culture exposure, increasing the risk of genetic instability [21].

Taking the above into account, the first stage of the experiment assessed the ability of leaf explants from individual somaclones to form callus and the number of regenerants produced (Figure 2B,C). All somaclones analyzed in this stage demonstrated a very high callus initiation capacity (Table 2), ranging from 95 to 100%. The highest callus tissue formation efficiency of 100% was observed for somaclones 5, 12, and 30, while the lowest was observed for somaclone 21. The highest number of regenerants developed from callus tissue was obtained for somaclone 6 (23), which may indicate its high regenerative competence, while the lowest was for somaclone 12 (7). Pasternak et al. (2024) [22] describe cytokinins such as BAP and kinetin as effective inducers of shoot proliferation. Numerous studies, particularly those by Jha et al. (2020) [23], emphasize that an important aspect of cytokinin action is the activation of key gene expression, such as WUSCHEL (WUS), which was shown to be essential during shoot regeneration. This leads to the transformation of callus cells and the initiation of shoot meristems. The absence of cytokinins often results in the regeneration process being halted at the undifferentiated callus stage, without shoot development. Therefore, in light of the obtained results, it should be concluded that for the species studied, the concentration of PGRs used effectively influenced the initiation of callus tissue with a diverse potential for regenerant formation.

### 3.2. Analysis of the Morphogenetic Potential of Regenerants

**Figure 3:**
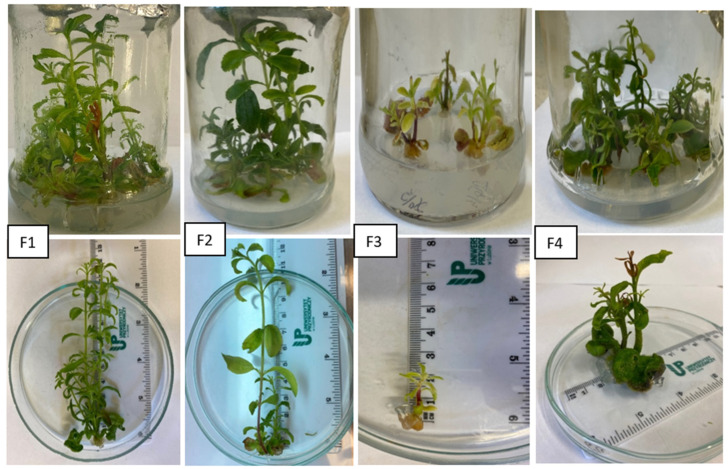
Comparison of the growth of the second-generation somaclones on the tested medium.

The essence of the analyses was to compare the regenerative potential of regenerants obtained from callus. Three basic parameters were assessed: the number of shoots obtained from a single explant, the length of the generated shoots, and the number of nodes for each shoot. The research presented in this publication is a continuation of the author’s earlier work on the analysis of somaclonal variability in *S. rebaudiana*. Indirect organogenesis was used again as a plant regeneration method to verify the hypothesis about the possibility of re-establishing somaclonal variability in regenerants or their genetic stability. The results of the regeneration process analyses on F1 medium with the addition of 0.5 mg/LBAP and 0.25 mg/Kinetin for six somaclones are presented in Table 3, Figure 3. The highest mean number of shoots was obtained for somaclone five regenerants, with a high diversity ranging from 4.0 to 12.0. This value differed significantly from the results obtained for the other regenerants. The lowest number of shoots—2.5—was recorded for somaclone 30 regenerants, with a low diversity range. Regenerants of somaclones 26, 30, 21, and 6 were characterized by the longest shoots and showed statistically significant differences in this respect compared to somaclone 12. For the mean number of nodes per shoot, no statistically significant differences were found between somaclones, while the diversity of this trait ranged from 1 to 5.

On F2 medium with 0.5 mg × dm^−3^ BAP, significant variation was observed between somaclone regenerants in terms of the analyzed traits (Table 4, Figure 3). The highest shoot number values were observed for somaclone six microplants, with a high range of their variation observed (2.0–11.0). Low values of this trait were recorded for somaclones 21 and 30, and somaclone 26 regenerants showed the lowest levels, but were also the genotypes with the highest average shoot length. The lowest average shoot length was observed for somaclone six genotypes, i.e., those with the highest average shoot number. Mean number of nodes per shoot values for somaclones ranged from 3.77 to 5.29, while for regenerants, the range was from 2 to 7.

The effect of the combination of phytohormones contained in F3 medium on the analyzed properties is presented in Table 5, Figure 3. The use of 1 mg × dm^−^^3^ BAP and 0.25 mg/Kinetin resulted in the inhibition of new shoot production, shortening their length, and a reduction in the mean number of nodes per shoot. No statistically significant differences were observed between somaclones for mean shoot length and mean number of nodes per shoot. However, with respect to the mean number of shoots, somaclone 26 regenerants had a significantly lower value compared to the other somaclones.

The use of F4 medium with the addition of 1 mg/LBAP did not significantly affect the diversification of the analyzed properties in the tested group of somaclones (Table 6, Figure 3). The highest value of the mean number of shoots was obtained by somaclone 21 regenerants (which also showed the highest mean shoot length), while the lowest was 6. The lowest mean number of nodes per shoot was characterized by somaclone 5 regenerants, for which the highest variability of the trait was observed in the range of 1 to 5.

Based on the analyses, it was found that for most of the studied somaclones, the greatest number of shoots were developed by explants grown in a medium with a low BAP concentration and supplemented with kinetin. Only for somaclone 6 was this process more efficient in a medium without kinetin. Increasing the BAP concentration to 1 mg/L reduced the efficiency of this process. Furthermore, a low BAP concentration in the medium (0.5 mg/L) had a beneficial effect on shoot length and the number of nodes per shoot Table S1. It should be noted that with BAP and kinetin contents of 1 and 0.25 mg/L in the medium, the values of all analyzed parameters were the lowest in virtually all somaclones, and only slightly higher values of traits could be observed after excluding kinetin from the medium. A similar response of the studied stevia somaclones (expressed by a similar range of variability of the analyzed traits) observed on F3 medium suggests a key role of BAP in the in vitro regeneration process of this species. It should therefore be concluded that in the studied species, WUS gene inactivation may likely be due to an above-optimal level of BAP, which on F3medium resulted in a very low number of regenerating shoots from explants in all somaclones analyzed in the experiment. Studies by Jadid et al. 2024 [24] also indicate that low concentrations of BAP and Kin in the medium result in a high shoot formation frequency, while high concentrations have the opposite effect. Razak et al. 2014 [25] emphasized this effect of the above-mentioned phytohormones, where, using nodal segment explants, a higher number of shoots was observed on media supplemented with 0.2 mg/L BAP compared to the same Kin concentration. Furthermore, with increasing BAP concentrations in the presence of Kin, the multiplication effect decreased. Aguirre-Medina et al. (2021) [26], on the other hand, reported a beneficial effect of BAP at a concentration of 0.5 mg/L on the number of shoots, their length, and the number of leaves. Moreover, the work of Rokosa and Kulpa (2020) [27] confirms that the length of multiplied shoots decreases when the BAP concentration is increased to 1 mg/L. The authors also demonstrated that the use of BAP at an optimal concentration not only promotes the formation of the most developed shoots but also influences significant leaf growth. Similarly, analysis of the results published by Ghose et al. (2022) [28] showed that although the addition of cytokinins increases the number of shoots, the use of only BAP at an appropriate concentration best supports the growth and development of stevia. Misal et al. (2024) [29], on the other hand, suggest that shoot regeneration from leaf explants can be effective on media containing BAP and auxin (IAA, IBA, NAA in various concentrations). Comparing the phenotypic variability of the studied somaclones with respect to the initial generation of somaclones, it should be stated that it has not changed in principle in relation to the features analyzed in the work. For example, the average number of shoots obtained for the six initial somaclones was 5.05, while the progeny generation was characterized by a very similar value of this trait at 4.98 [30]. Furthermore, the range of its maximum and minimum values was also not exceeded. On the other hand, considering the values of this trait for individual somaclones, it should be noted that it increased in two and decreased in four of them. In relation to the other two properties, their average values were lower than those of the initial somaclones. However, within the somaclone, exceptionally favorable regenerants can be identified, e.g., within somaclone 6, regenerant 6/3 exceeded the average shoot length of the analyzed somaclone group by more than twice. As the above data suggest, somaclonal variation may be stable to some extent, but it is not always stable. This suggests the need for thorough genetic and phenotypic studies over several generations of regenerants.

### 3.3. SCoT Markers—Genotype Analysis

Many molecular techniques exist for detecting genetic variation within somaclones and comparing them to the donor plant using DNA analysis. One such technique is the start codon-targeted polymorphism (SCoT) detection system. This system allows for the detection of modifications occurring in coding sequences, even those that do not alter the gene product to an extent observable in the phenotype [31]. This marker system was used in this study because it is a continuation of the work by Siemińska et al., 2024 [16]. The primer set used in the cited study and in this study allows for reliable cross-referencing of the obtained results with the results of the previous experiment. SCoT is a simple and reliable dominant marker, and unlike RAPD or ISSR, which are based on non-coding regions of the genome, the SCoT technique correlates with functional genes and their corresponding traits. The multilocus nature of the marker is helpful in identifying high genetic polymorphism. In addition, the use of long primers and high annealing temperature enhances the reproducibility of SCoT primers [32]. Being associated with the initiation code, SCoT markers are abundant in the genome and provide extensive genetic information [33].

**Figure 4:**
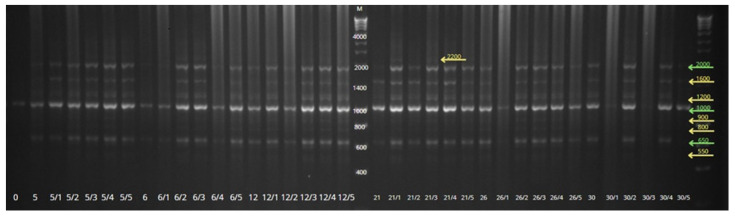
SCoT fingerprints of thirty-seven stevia genotypes using SCoT 75 primer. M—standard of DNA fragment size. Polymorphic bands are marked in yellow, monomorphic bands are marked in green.

In the present study, a total of 11 SCoT primers produced 70 scorable DNA fragments ranging in size from 220 to 4000 bp (Table 7). The amplicons observed for the SCoT marker were monomorphic and polymorphic in thirty regenerants, six somaclones, as well as the mother plant. The average of DNA bands per primer was 6.4. The number of amplicons per primer varied from 3 (SCoT 23, SCoT 33, and SCoT 46) to 9 (SCoT 21,75,83) bands. Figure 4 shows an example of banding patterns obtained from SCoT (SCoT-75); banding patterns of the remaining primers are provided in the Supplementary Materials—Figures S1–S10.

Since Krishna et al., 2006 [37] pointed out the significant influence of genotype on the regeneration process, citing literature data to compare the results obtained by researchers at the phenotypic or genotypic level, one very important factor should be considered: the origin of the plant material used for research, as it determines the genetic variability or homogeneity of the explants, which results in the variability or homogeneity of the regenerants. Recently published research works related to the topic of regeneration of this species in vitro report the use of explants from genotypes L020, Morita II, and L102 [38], single cultivated genotype of *S. rebaudiana* [39], soil-grown 30-day-old seedlings of stevia [28] and general *S. rebaudiana* accession Mini [24], while there are no reports of their molecular analysis. In this study, the plant material (somaclones) used to initiate the in vitro culture was analyzed at the DNA level, which allowed us to determine the existence of genetic diversity between the studied somaclones. Obtaining the next generation of somaclones and analyzing them with SCoT markers enabled us to assess their genetic stability. Based on the results obtained in this study, the studied group of somaclones exhibits a higher degree of polymorphism (55.2%) compared to the initial group, which had 33.7% [16]. This indicates that indirect regeneration using callus tissue can result in somaclones characterized by increased genetic diversity.

**Figure 5:**
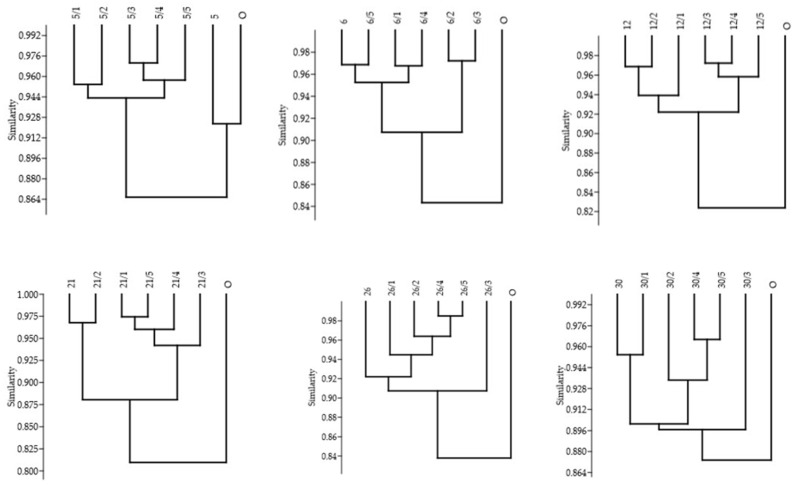
Dendrogram illustrating the grouping of the tested first and second generation somaclones and the donor plant.

However, the analysis of genetic similarity of somaclones presented in the form of individual dendrograms (cluster analysis performed using the UPGMA method) indicates that in most cases, greater genetic diversity was revealed as a result of indirect regeneration in the first generation of somaclones compared to the second (Figure 5). This results from the fact that all 5 analyzed somaclones and the first-generation somaclones were first combined into one large cluster, and the donor plant (0) was added last, as observed for somaclones 6, 12, 21, 26, and 30. Furthermore, the formation of two clearly separate clusters suggests the existence of greater potential for revealing genetic changes in explants derived from the original plant, including insertions, deletions, point mutations, chromosomal rearrangements, and changes in ploidy level, as noted by Ferreira et al., 2023 [40]. The range of changes revealed results from the complex impact of the in vitro culture environment on the explant cells and reflects the diversity of alleles in the functional regions of the genome within the loci scanned using the SCoT marker system. Hesami et al., 2023 [41] and Miryeganeh and Armitage 2025 [42] identify the following as the main factors influencing the nature of these changes: genotype, explant type, medium composition, phytohormone concentration, passage number, and culture age. In the case of somaclone 5, the donor plant and its derived somaclone were observed to cluster together in one cluster, and the remaining five in a second cluster, which in turn indicates relatively small genetic differences between second-generation somaclones. According to Majmunder et al., 2025 [21], different genotypes may have different potential to reveal variability under in vitro culture conditions.

## 4. Conclusions

Thus, indirect organogenesis allows for the production of subsequent generations of genetically unstable somaclones, creating the potential for obtaining new phenotypic variants useful in plant breeding. The results of this study indicate that the use of indirect organogenesis reveals somaclonal variation within second-generation regenerants. The SCoT marker system is an effective tool for assessing the degree of genetic diversity within the studied regenerant population. These markers, targeting conserved initiation codon (ATG) sequences, allow for the detection of DNA polymorphisms in functional regions of the genome and are particularly useful in the analysis of plants with limited available genomic data, such as *S. rebaudiana*. Therefore, combining plant regeneration techniques via indirect organogenesis with molecular analysis using SCoT markers provides an effective approach for studying intraspecific genetic diversity in stevia induced in vitro, which may have significant implications for both breeding programs and the conservation of this species’ genetic resources.

## Figures and Tables

**Table 1:** Concentration of plant growth regulators used in the media used to multiply regenerants.

	PGR	BAP (mg × dm^−3^) (Benzylaminopurine)	Kin (mg × dm^−3^) (Kinetin)
Medium			
F1		0.5	0.25
F2		0.5	-
F3		1	0.25
F4		1	-

**Table 2:** Evaluation of the morphogenetic potential of callus tissue.

No. of Somaclone	Number of Explants	Percentage of Explants Producing Callus	Number of Regenerants
5	100	100	15
6	100	98	23
12	100	100	7
21	100	95	12
26	100	97	10
30	100	100	13

**Table 3:** Diversity of morphogenetic abilities of second-generation somaclones obtained on F1 medium.

No. of Somaclone	F1 Medium		
	Mean Number of Shoots	Mean Shoot Length (cm)	Mean Number of Nodes per Shoot
5	8.00 a * (4.0–12.0)	3.40 bc (2.5–4.5)	3.31 a (2.0–4.0)
6	3.71 cd (2.0–5.0)	4.14 ab (0.7–8.0)	3.06 a (1.0–5.0)
12	5.78 b (4.0–7.0)	2.59 c (2.2–3.0)	2.93 a (2.0–4.0)
21	4.65 bc (3.0–6.0)	4.17 ab (2.9–5.8)	2.92 a (2.0–3.0)
26	5.29 b (2.0–8.0)	4.82 a (4.1–5.5)	2.81 a (2.0–4.0)
30	2.50 d (2.0–3.0)	4.58 a (3.1–6.2)	3.00 a (2.0–4.0)

* Means following the same letter within columns are not significantly different according to Duncan’s test (*p* < 0.05), numbers in parentheses represent the range of trait values.

**Table 4:** Diversity of morphogenetic abilities of second-generation somaclones obtained on F2 medium.

No. of Somaclone	F2 Medium		
	Mean Number of Shoots	Mean Shoot Length (cm)	Mean Number of Nodes per Shoot
5	4.22 b * (2.0–5.0)	6.11 ab (3.3–10.2)	4.75 ab (3.0–5.0)
6	6.19 a (2.0–11.0)	4.61 b (3.7–5.4)	3.77 b (2.0–5.0)
12	3.08 c (1.0–4.0)	5.04 b (2.5–7.1)	4.83 ab (3.0–6.0)
21	2.82 cd (1.0–4.0)	5.20 b (2.2–7.1)	3.81 b (2.0–6.0)
26	1.82 d (1.0–2.0)	7.00 a (4.6–10.3)	5.29 a (4.0–7.0)
30	2.84 cd (2.0–3.0)	5.98 ab (4.3–7.4)	4.80 ab (3.0–6.0)

* Means following the same letter within columns are not significantly different according to Duncan’s test (*p* < 0.05), numbers in parentheses represent the range of trait values.

**Table 5:** Diversity of morphogenetic abilities of second-generation somaclones obtained on F3 medium.

No. of Somaclone	F3 Medium		
	Mean Number of Shoots	Mean Shoot Length (cm)	Mean Number of Nodes per Shoot
5	1.00 a * (1.0–1.0)	1.80 a (1.00–2.50)	2.00 a (1.0–3.0)
6	1.00 a (1.0–1.0)	1.90 a (1.25–2.75)	2.50 a (1.0–4.0)
12	1.86 a (1.0–3.0)	2.07 a (1.00–2.50)	2.71 a (2.0–3.0)
21	1.00 a (1.0–1.0)	1.95 a (1.50–2.50)	1.90 a (1.0–2.0)
26	0.90 b (0.0–1.0)	1.95 a (1.25–2.50)	2.90 a (2.0–3.0)
30	1.00 a (1.0–1.0)	1.94 a (1.25–2.25)	2.70 a (2.0–3.0)

* Means following the same letter within columns are not significantly different according to Duncan’s test (*p* < 0.05), numbers in parentheses represent the range of trait values.

**Table 6:** Diversity of morphogenetic abilities of second-generation somaclones obtained on F4 medium.

No. of Somaclone	F4 Medium		
	Mean Number of Shoots	Mean Shoot Length (cm)	Mean Number of Nodes per Shoot
5	2.13 a * (1.0–3.0)	2.86 a (2.50–3.00)	2.87 a (1.0–5.0)
6	2.11 a (1.0–3.0)	2.83 a (2.00–3.50)	3.11 a (2.0–3.0)
12	1.86 a (1.0–3.0)	4.43 a (2.00–5.83)	4.43 a (3.0–6.0)
21	3.00 a (1.0–3.0)	4.96 a (1.80–7.10)	4.14 a (3.0–5.0)
26	1.89 a (1.0–2.0)	3.56 a (2.75–4.33)	3.11 a (2.0–4.0)
30	2.38 a (1.0–3.0)	4.57 a (3.00–5.33)	4.00 a (2.0–5.0)

* Means following the same letter within columns are not significantly different according to Duncan’s test (*p* < 0.05), numbers in parentheses represent the range of trait values.

**Table 7:** Starter sequence [34,35,36] and molecular polymorphism analyzed by SCoT markers.

No. of Primer	Starter Sequence 5′-3′	Number of Products			Percentage of Polymorphism %	Size Range (bp)
		Monomorphic	Polymorphic	Total		
2	CAACAATGGCTACCACCC	2	4	6	67	1000–4000
4	CAACAATGGCTACCACCT	4	2	6	33	1400–3800
21	ACGACATGGCGACCCACA	5	4	9	44	370–2750
23	CACCATGGCTACCACCAG	2	1	3	33	1300–1700
28	CCATGGCTACCACCGCCA	7	2	9	22	220–2500
30	CCATGGCTACCACCGGCG	1	4	5	80	2250–4000
33	CCATGGCTACCACCGCAG	0	3	3	100	750–1000
46	ACAATGGCTACCACTGAG	1	2	3	67	900–4000
75	CCATGGCTACCACCGGAG	3	6	9	67	550–2200
83	ACGACATGGCGACCAGCG	4	5	9	56	500–2500
90	CCATGGCTACCACCGGCA	5	3	8	38	500–3000
Total	-	34	36	70	-	220–4000
Average/ primer	-	3.1	3.3	6.4	55.2	-

## Data Availability

The original contributions presented in this study are included in the article/supplementary material. Further inquiries can be directed to the corresponding author.

## Supplementary Materials

The following supporting information can be downloaded at https://www.mdpi.com/article/10.3390/genes16101203/s1, Figures S1–S10: SCoT fingerprints of stevia genotypes using SCoT 2, 4, 21, 23, 28, 30, 33, 46, 83, and 90 primers. M—standard of DNA fragment size. Polymorphic bands are marked in yellow, monomorphic bands are marked in green. Table S1: Diversity of morphogenetic abilities of second-generation somaclones obtained on tested media.

## Abbreviations

The following abbreviations are used in this manuscript:

SGs	steviol glycosides (SGs)
MS	Murashige and Skoog
BAP	6-benzylaminopurine
NAA	1-naphthaleneacetic acid
2,4-D	2,4-dichlorophenoxyacetic acid
Kin	Kinetin
SCoT	Start Codon Targeted
WUS	WUSCHEL
