# Supplementary material for: In Vitro Regeneration of Stevia rebaudiana Bertoni Using Somaclonal Variation as a Tool for Genetic Diversification
Source: Genes (Basel). 2025 Oct 14;16(10):1203. doi: 10.3390/genes16101203 (PMC12562407; doi:10.3390/genes16101203)
Supplement: Supplementary file 1 [file genes-16-01203-s001.zip › Table S1.pdf]

Table S1. Diversity of morphogenetic abilities of second generation somaclones obtained on tested media.

Medium	Mean number of shoots	Mean shoot length (cm)	Mean number of nodes per shoot
F1	5.40a	3.82b	3.01c
F2	4.12b	5.40a	4.40a
F3	1.10d	1.94c	2.48d
F4	2.34c	4.09b	3.67b
