# Supplementary figures and images for: In Vitro Regeneration of Stevia rebaudiana Bertoni Using Somaclonal Variation as a Tool for Genetic Diversification
Source: Genes (Basel). 2025 Oct 14;16(10):1203. doi: 10.3390/genes16101203 (PMC12562407; doi:10.3390/genes16101203)

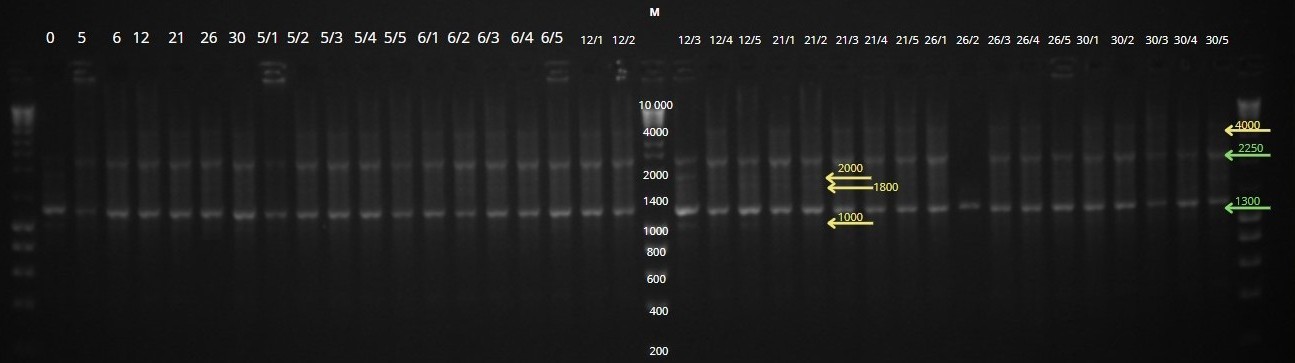

Supplement: Supplementary file 1 [file genes-16-01203-s001.zip › FigureS1.jpg]

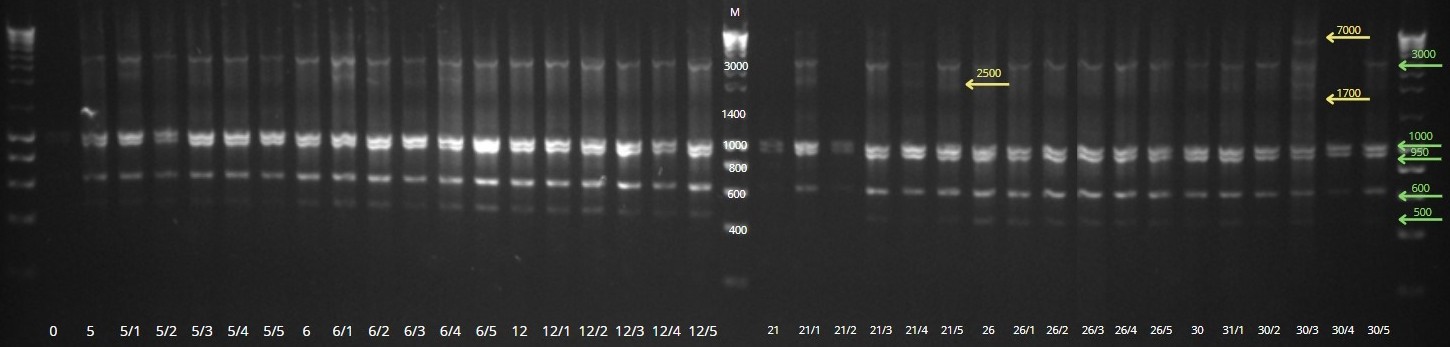

Supplement: Supplementary file 1 [file genes-16-01203-s001.zip › FigureS10.jpg]

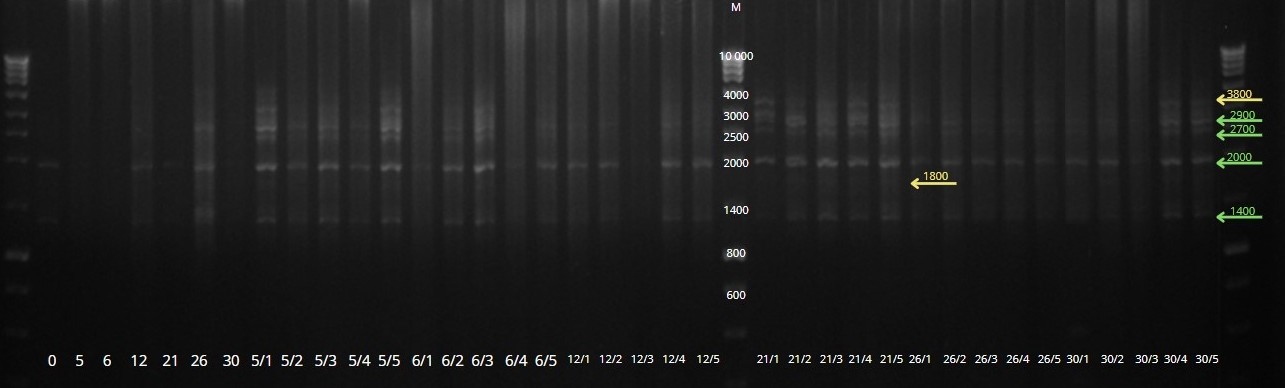

Supplement: Supplementary file 1 [file genes-16-01203-s001.zip › FigureS2.jpg]

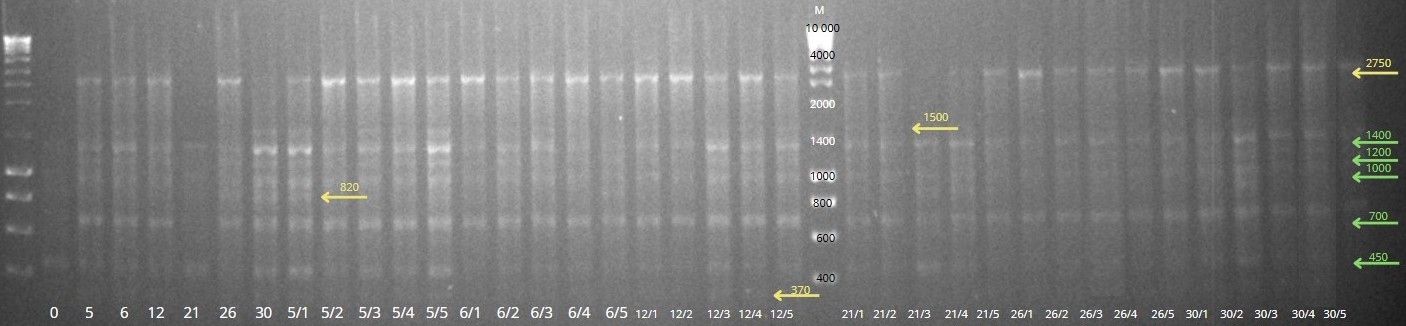

Supplement: Supplementary file 1 [file genes-16-01203-s001.zip › FigureS3.jpg]

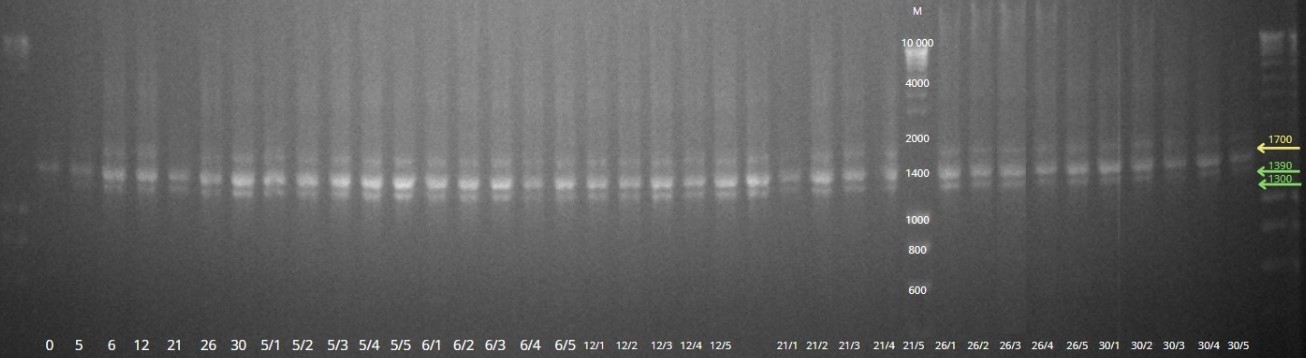

Supplement: Supplementary file 1 [file genes-16-01203-s001.zip › FigureS4.jpg]

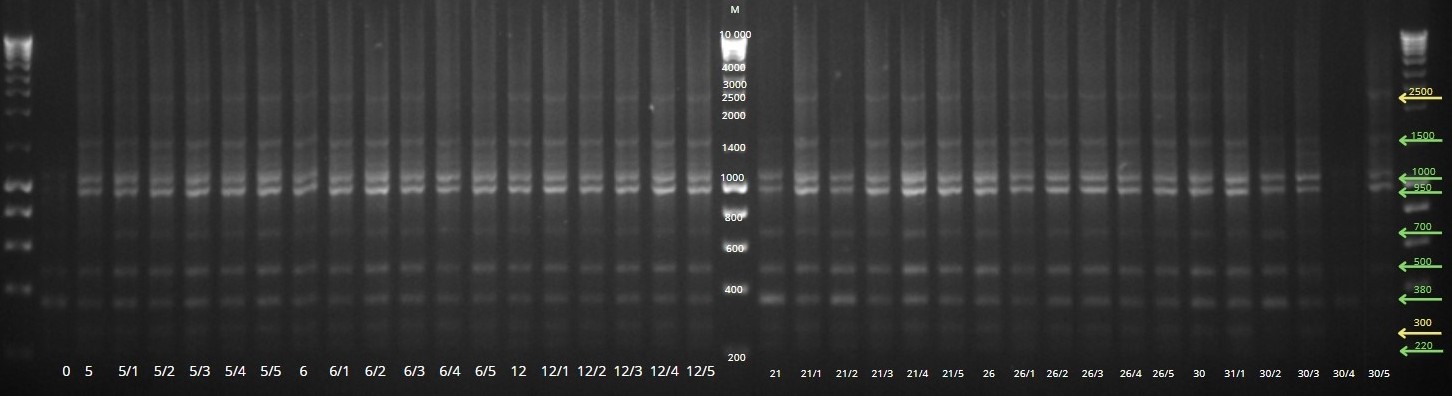

Supplement: Supplementary file 1 [file genes-16-01203-s001.zip › FigureS5.jpg]

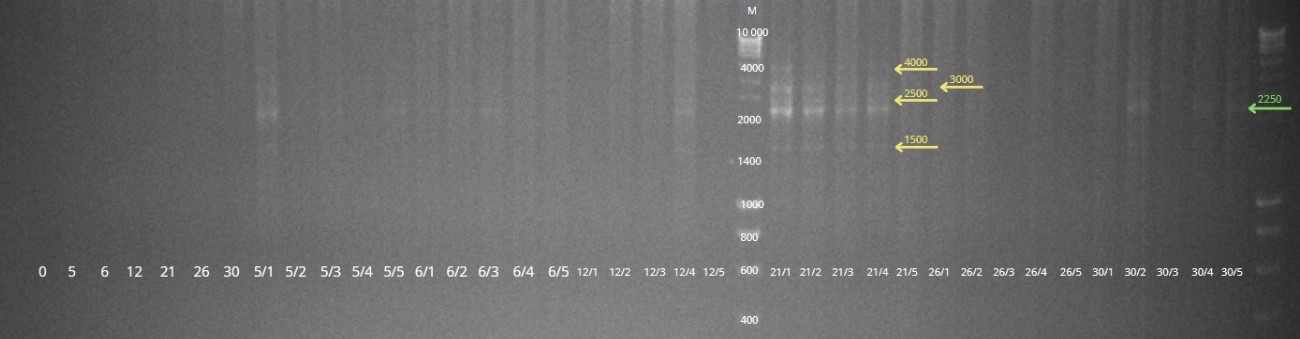

Supplement: Supplementary file 1 [file genes-16-01203-s001.zip › FigureS6.jpg]

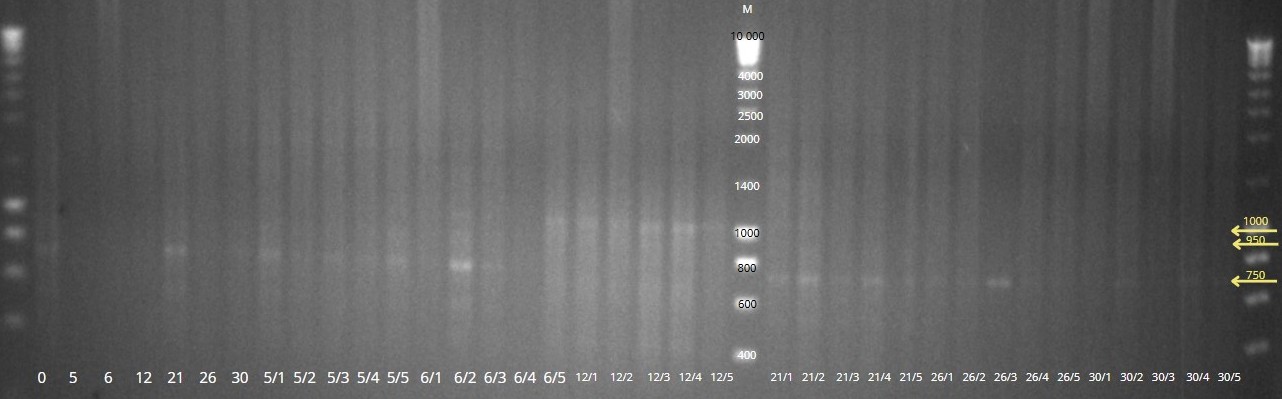

Supplement: Supplementary file 1 [file genes-16-01203-s001.zip › FigureS7.jpg]

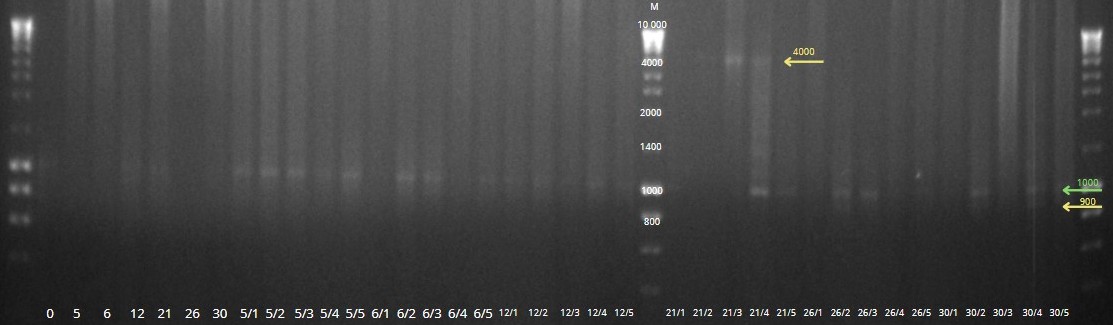

Supplement: Supplementary file 1 [file genes-16-01203-s001.zip › FigureS8.jpg]

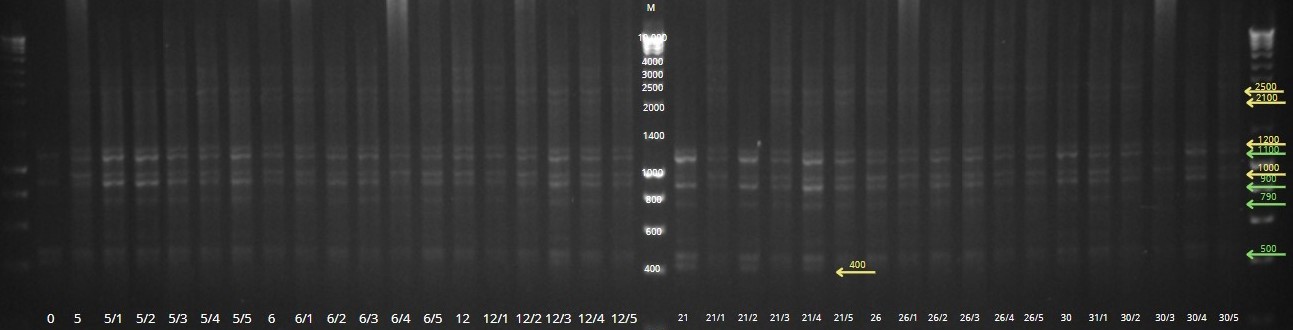

Supplement: Supplementary file 1 [file genes-16-01203-s001.zip › FigureS9.jpg]
